# Supplementary material for: New-to-nature CO2-dependent acetyl-CoA assimilation enabled by an engineered B12-dependent acyl-CoA mutase
Source: Nat Commun. 2024 Nov 26;15:10235. doi: 10.1038/s41467-024-53762-9 (PMC11599936; doi:10.1038/s41467-024-53762-9)
Supplement: Supplementary file 1 — Supplementary Information [file 41467_2024_53762_MOESM1_ESM.pdf]

New-to-nature CO_2-dependent acetyl-CoA assimilation enabled by an engineered B_{12}-dependent acyl-CoA mutase

Schulz-Mirbach, Wichmann, Satanowski *et al.*

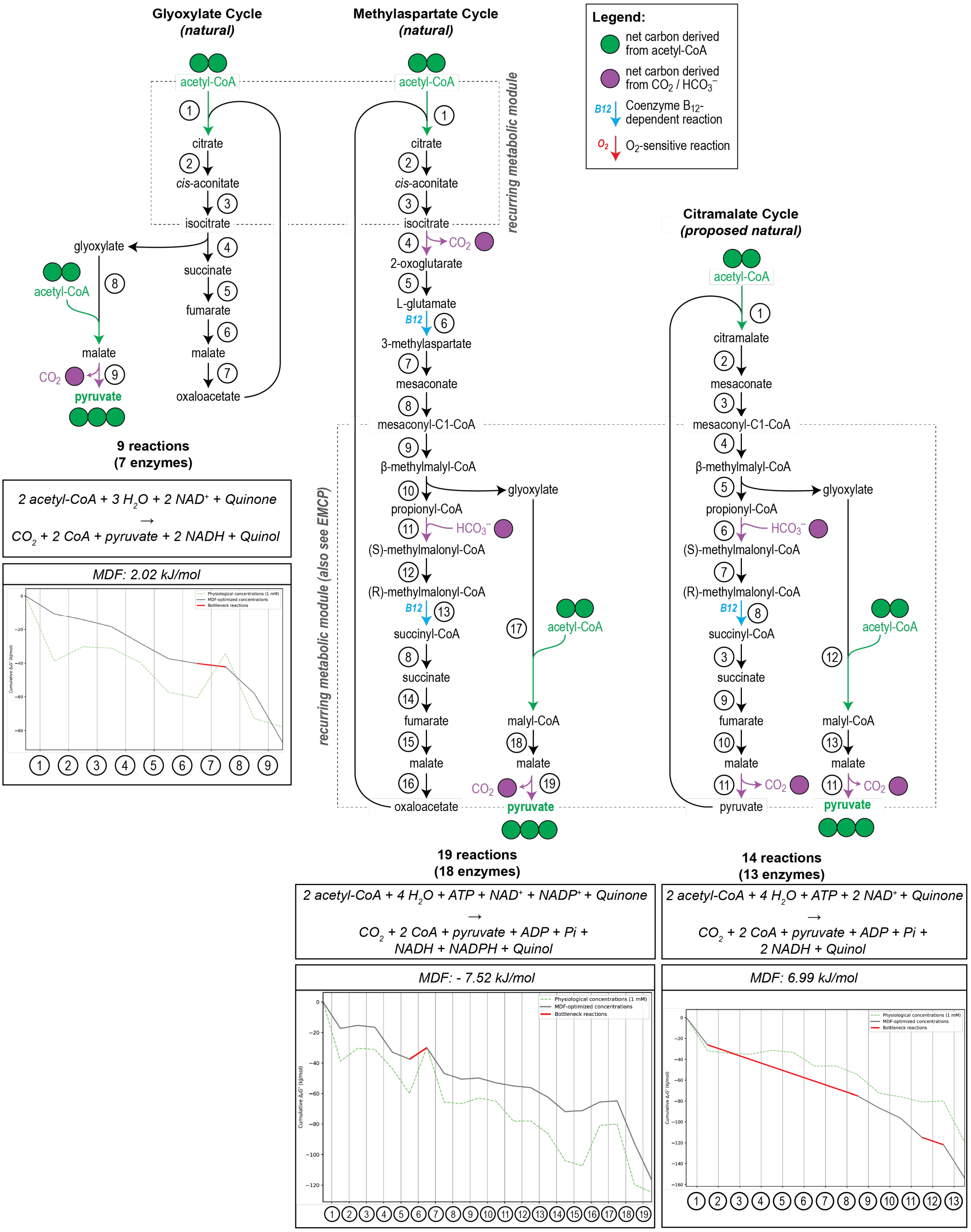

Supplementary Figure 1. The natural diversity of metabolic pathways for carbon-neutral assimilation of acetyl-CoA into the central C$_3$ intermediate pyruvate. The carbon coloring describes the origin of carbon atoms in the final product, indicating only the net stoichiometry regarding carboxylation/decarboxylation steps (not the specific position of these atoms such as e.g. observable via ^{13}C-tracing experiments). For clarity, co-factors such as ATP and reducing equivalents are omitted. Max-Min driving force (MDF) analysis of thermodynamic feasibility was performed using equilibrator [1] (see Methods).

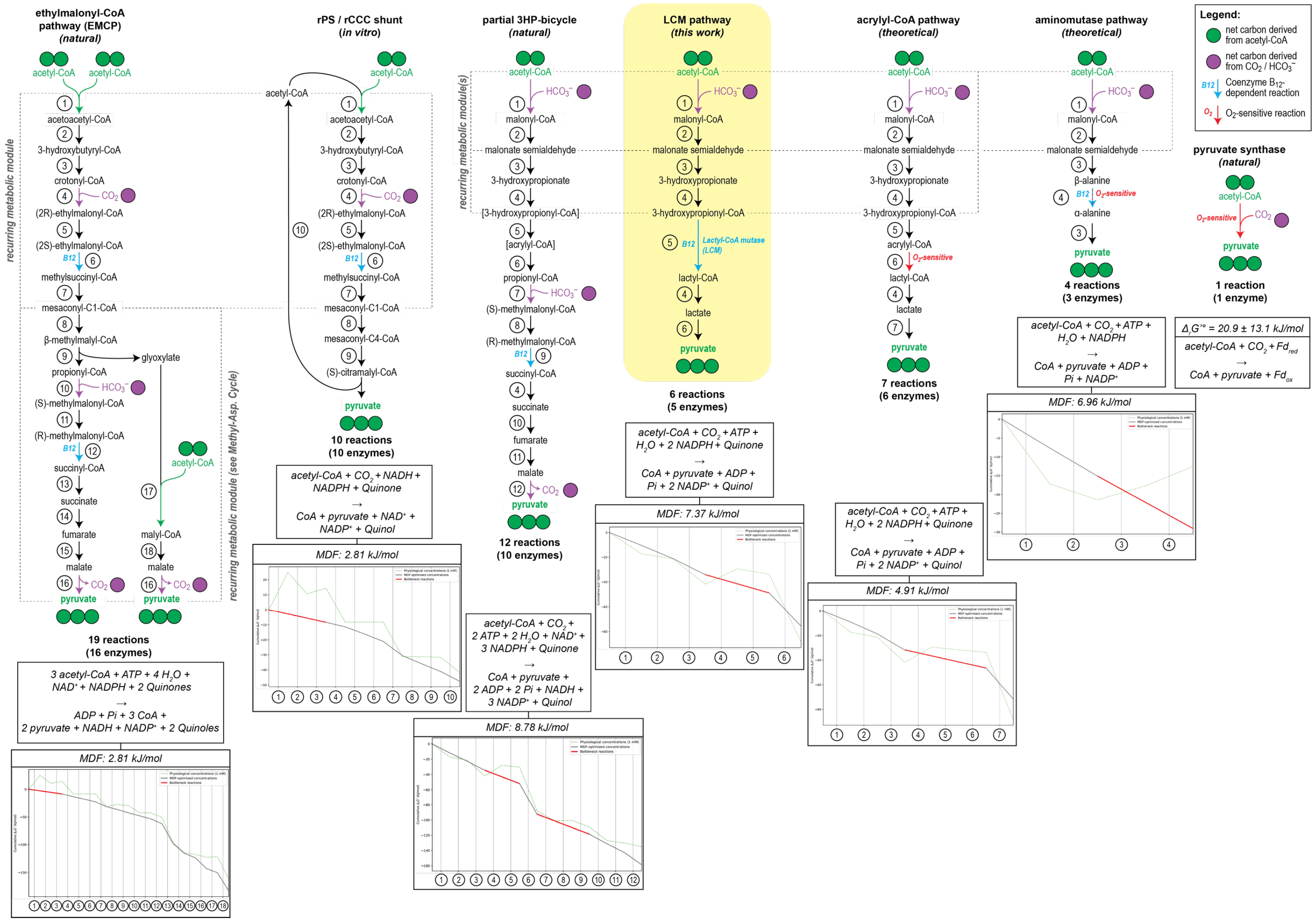

Supplementary Figure 2. Reductive acetyl-CoA assimilation routes. This scheme shows selected natural and synthetic carbon-negative (CO_2-assimilating) pathways for conversion of acetyl-CoA intothe C_3-compound pyruvate. The carbon coloring describes the origin of carbon atoms in the final product, indicating only the net stoichiometry regarding carboxylation/decarboxylation steps (not the specific position of these atoms such as e.g. observable via ^{13}C-tracing experiments). For clarity, co-factors such as ATP and reducing equivalents are omitted. The rPS shunt (reductive pyruvate synthesis) has been proposed and analyzed computationally [2] and demonstrated *in vitro* [3]. The "acrylyl-CoA pathway" and "aminomutase pathway" have been proposed in variants of the malonyl-CoA-oxaloacetate-glyoxylate (MOG) cycles [4]. (Co-)Assimilation of acetyl-CoA via part of the 3HP-bicycle has been described in [5]. Max-Min driving force (MDF) analysis of thermodynamic feasibility was performed using equilibrator [1] (see Methods).

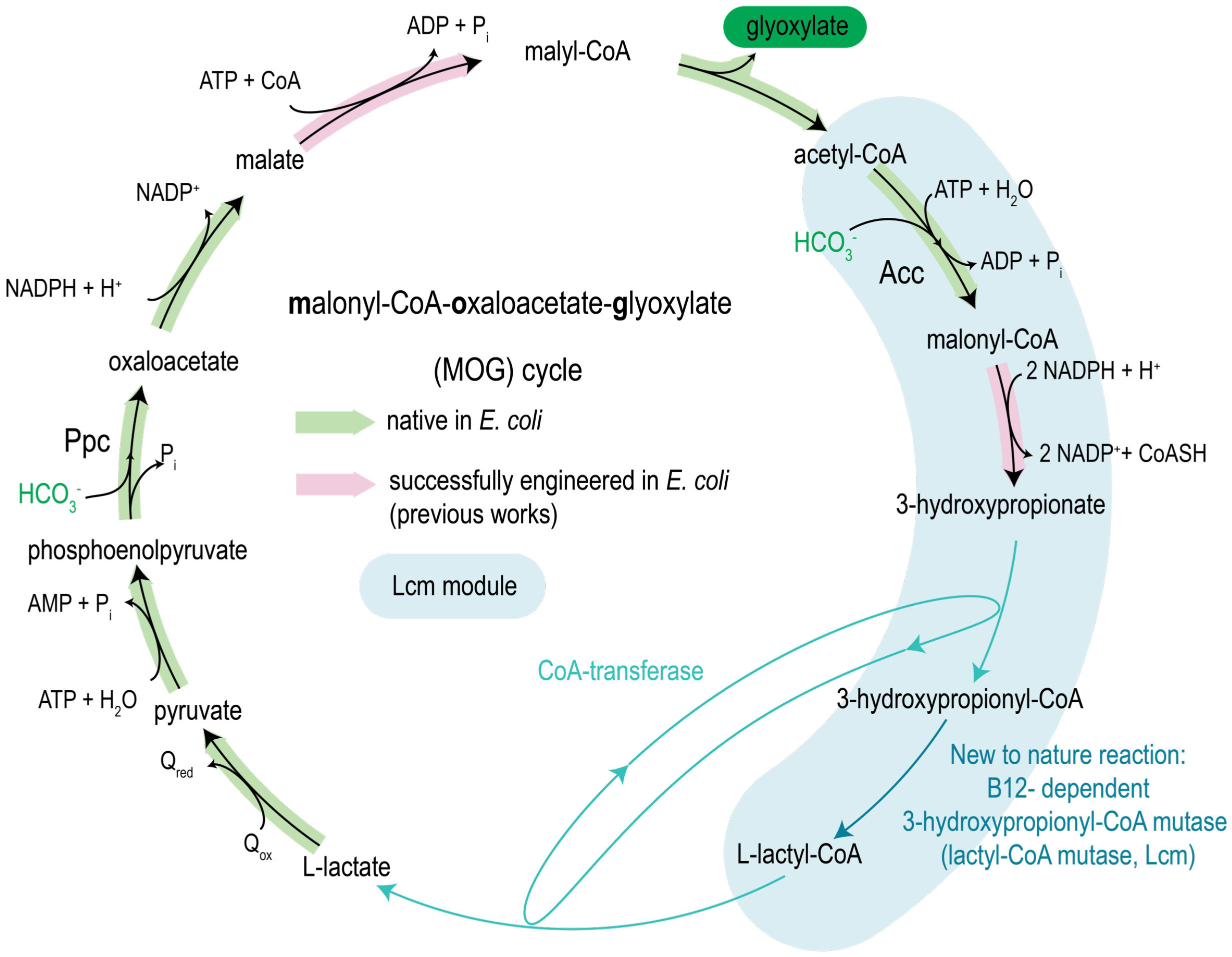

Supplementary Figure 3. A lactyl-CoA mutase dependent malonyl-CoA-oxaloacetate-glyoxylate (MOG) cycle. The conversion of 3-hydroxypropionate to lactate via the novel lactyl-CoA mutase reaction and a CoA transferase could allow pyruvate regeneration from acetyl-CoA. After phosphoenolpyruvate is carboxylated, malate activation to malyl-CoA and a subsequent lyase reaction can generate the cycle product glyoxylate and acetyl-CoA, the substrate for carboxylation by acetyl-CoA carboxylase. Reactions underlayed with light green are native *E. coli* reactions, light pink underlays indicate successful implementation in *E. coli* in previous works. The Lcm module, including the novel CoA-transferase (turquoise) and mutase (teal) reaction sequence is highlighted by the blue box.

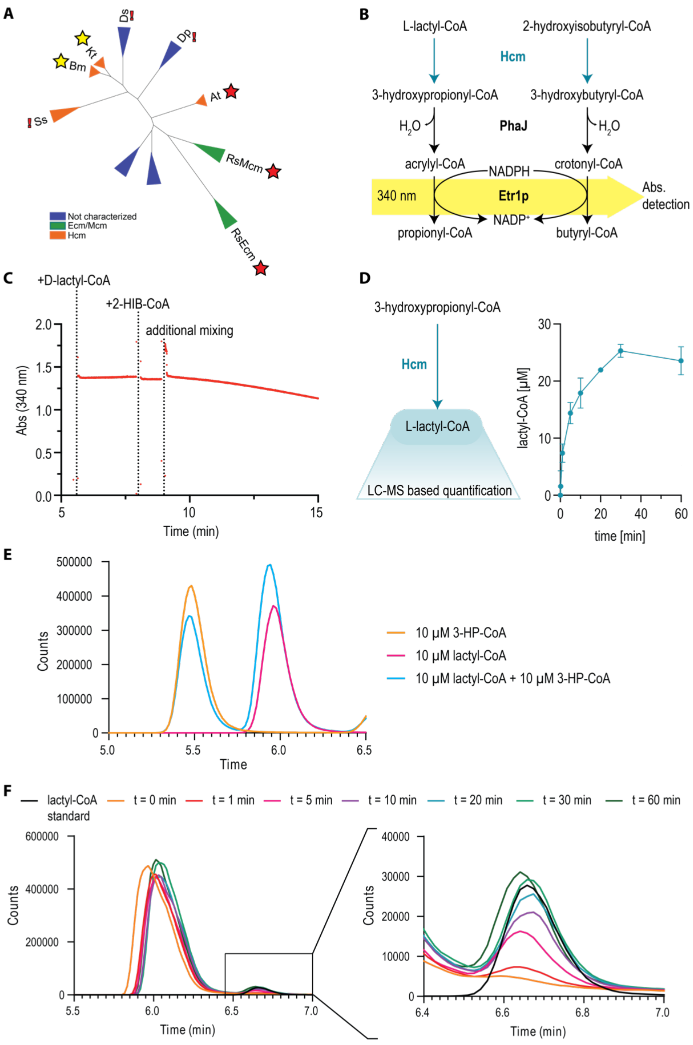

Supplementary Figure 4. Identification of scaffold enzymes with lactyl-CoA mutase (Lcm) activity. **A)** Phylogenetic tree of chosen candidate enzymes using MUSCLE [6] and FastTree2 [7]. Large subunits were used to construct the tree. Four hydroxybutyryl-CoA mutases (Hcm), two ethylmalonyl-CoA mutases (Ecm) or methylmalonyl-CoA mutases (Mcm) and four uncharacterized B12-dependent mutases were chosen for *in vitro* testing. The large subunits (A subunit) of the following putative mutases could not be purified (red exclamation mark): NCBI accessions HBB18459.1 (SsHcmA), HBB18460.1 (SsHcmB)), HIJ39843.1 (DpHcmA), HIJ39844.1 (DpHcmB), MAF34037.1 (DsHcmA) and MAF34038.1 (DsHcmB). Mutase variants marked with a red star were tested and had no activity with lactyl-CoA, variants with a yellow star had promiscuous activity with lactyl-CoA. **B)** Photometric assay to test Lcm and Hcm activity *in vitro*. The enoyl-CoA hydratase PhaJ and the enoyl thioester reductase Etr1p were used as coupling enzymes to follow NADPH consumption as readout for Lcm / Hcm activity. **C)** Activity confirmation for the *Aquincola tertiaricarbonis* mutase using the coupled assay shown in panel B. NADPH consumption occurs only once 2-hydroxyisobutyryl-CoA is added, for which a turnover of ~ 1.5 min^{-1} is observed. **D)** Lactyl-CoA formation from 3-HP-CoA by the *B. massiliosenegalensis* mutase (wild type). L-lactyl-CoA formation over time was quantified by LC-MS. Mean values of n=3 are shown with standard deviation. **E)** Chromatographic separation of lactyl-CoA and 3-HP-CoA. 10 µM standards were used as indicated by the legend. **F)** Chromatographic detection of lactyl-CoA formation from 3-HP-CoA via the LCM over time (see panel D). Source data are provided as Source Data file.

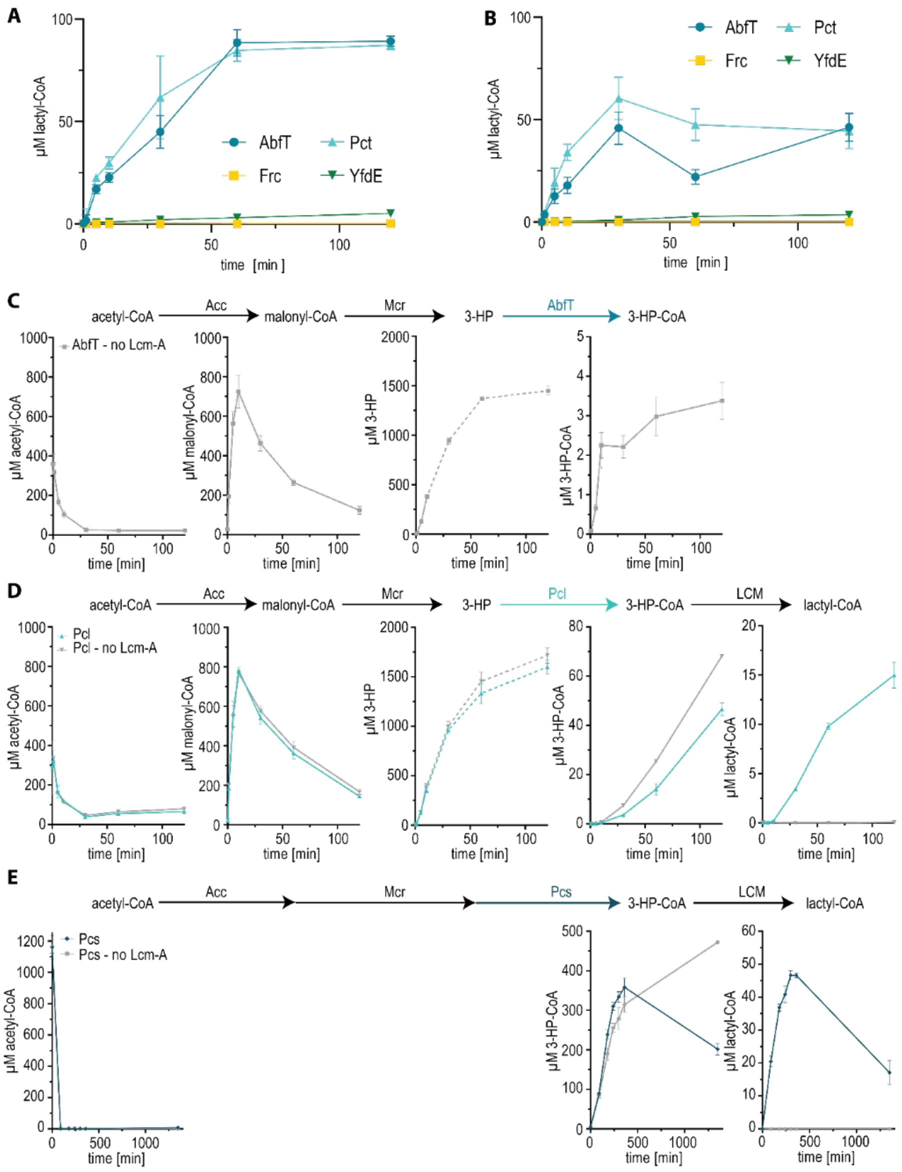

Supplementary Figure 5. Assembly and optimization of the Lcm module. A) Testing of CoA transferase candidates for the formation of lactyl-CoA from lactate with acetyl-CoA as CoA source. Tested were AbfT (teal), Pct (turquoise), Frc (yellow) or YfdE (green). Mean values of n=3 are shown with standard deviation in all panels. **B)** Lactyl-CoA formation from lactate with 3-hydroxypropionyl-CoA as CoA source, catalyzed by the CoA transferases AbfT (teal), Pct (turquoise), Frc (yellow) or YfdE (green). **C)** 3-HP-CoA formation from 1 mM acetyl-CoA and 2 mM acetate via the partial Lcm module with AbfT as CoA transferase. 3-HP concentrations with dotted lines are outside of the linear measurement range, but were included as confirmation for 3-HP synthesis. **D)** Lactyl-CoA formation from 1 mM acetyl-CoA via the Lcm module with Pcl for 3-hydroxypropionyl-CoA synthesis from 3-HP. 3-HP concentrations with dotted lines are outside of the linear measurement range, but were included as confirmation for 3-HP synthesis. **E)** Lactyl-CoA formation from 1 mM acetyl-CoA with Pcs for 3-HP-CoA synthesis. Due to the similarity of the results for AbfT and Pcl, malonyl-CoA and 3-HP formation was not quantified again. Note that the measurement period was extended to 22.5 hours (1350 minutes) this time. Source data are provided as Source Data file.

A

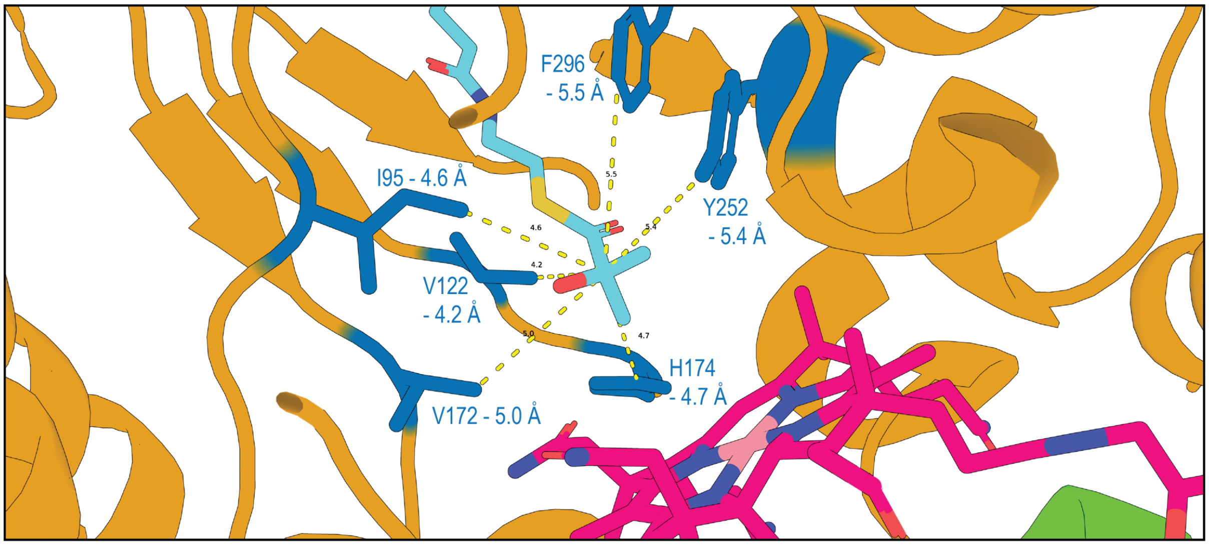

B

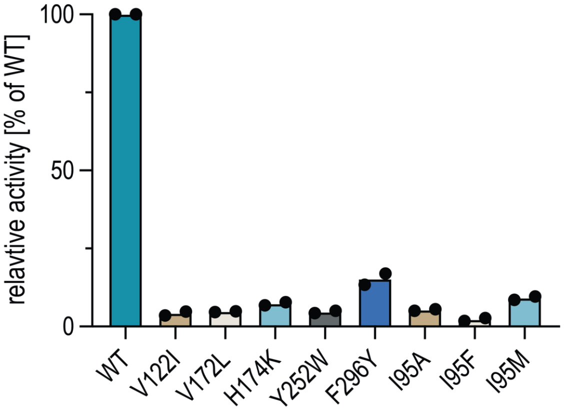

Supplementary Figure 6: Rational engineering of the mutase reduced Lcm activity. A) Single amino acid mutations were introduced with the intent of narrowing the active site to improve the activity with lactyl-CoA. We chose residues that we expected to surround the acyl-function of the CoA thioester with about 5 Å distance according to the AlphaFold2 prediction of the Lcm structure (colored in blue, the respective distance of chosen residues to 2-HIB-CoA is indicated next to each residue). With this, we aimed to restrict the space available so lactyl-CoA could bind better. We determined CoA thioester binding sites by using the published crystal structure of the *A. tertiaricarbonis* homologue as template (PDB: 4r3u). The B_{12} cofactor is colored magenta. **B)** All rationally designed mutant variants had much lower Lcm activity compared to the wild-type variant (k_{cat} of 1.8 min^{-1}). Shown is the relative activity in percent (variant turnover compared to wild type turnover) in the 3-hydroxypropionyl-CoA forming reaction direction. 1.5 mM L-lactyl-CoA was used with 5 µM of each subunit with 12.5 µM of coenzyme B_{12} at 30°C (n=2). Source data are provided as Source Data file.

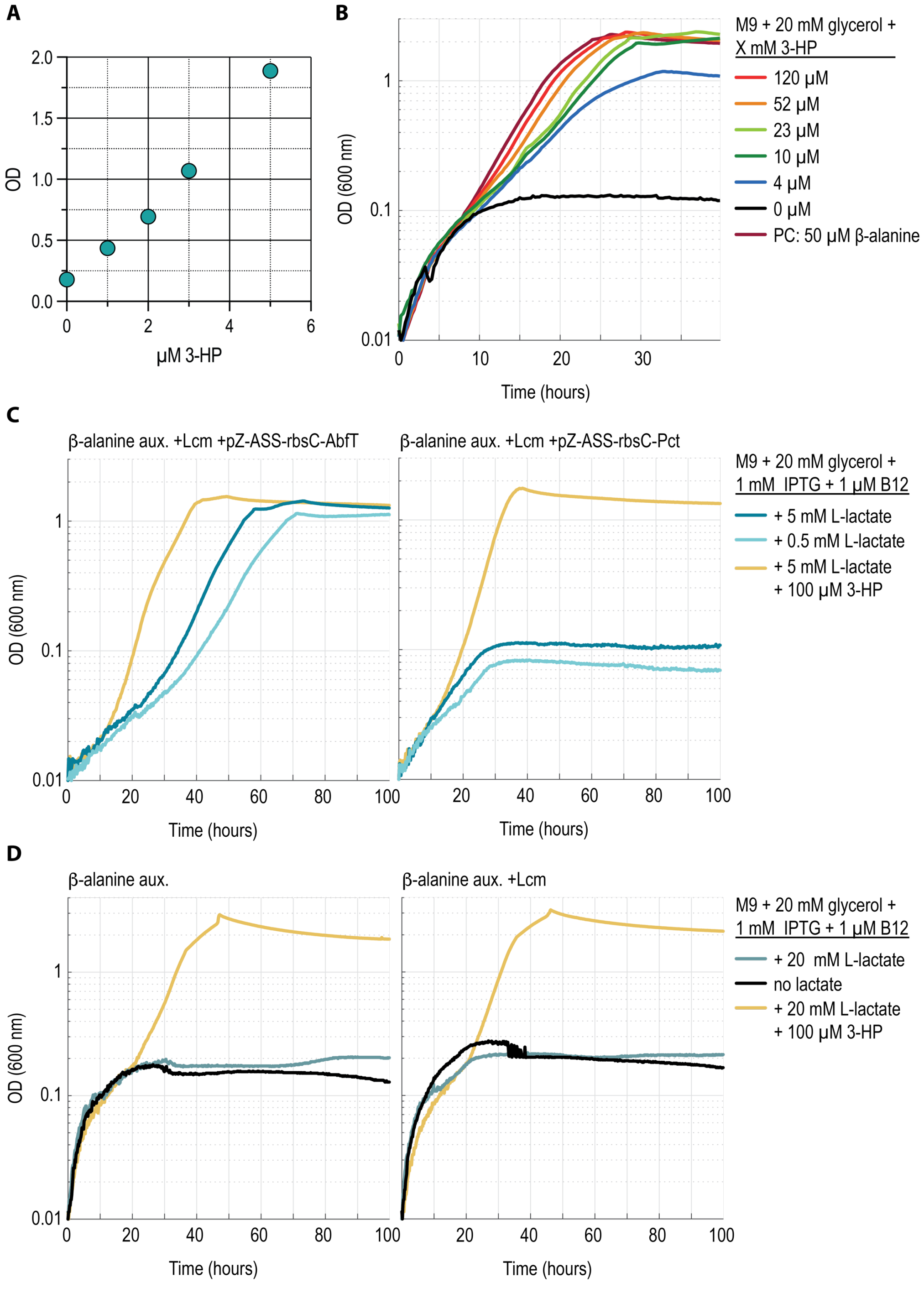

Supplementary Figure 7. CoA transferase performance evaluation *in vivo*. A) Maximum optical density achieved by the β-alanine auxotrophic 3-HP sensor strain in M9 + 20 mM glycerol with different 3-HP concentrations. The experiment was performed in tubes (n=1). **B)** Growth of the the β-alanine auxotrophic strain with various 3-HP concentrations. **C)** The CoA transferase AbfT (left graph) allows growth of the β-alanine auxotrophic strain with L-lactate instead of 3-hydroxypropionate. In contrast, Pct expression results in much lower biomass yields with L-lactate instead of 3-HP than the expression of AbfT (right graph). **D)** The β-alanine auxotroph without additional plasmids (left) or with only the Lcm (right graph) can only grow once 3-HP is provided (yellow curves). Source data are provided as Source Data file.

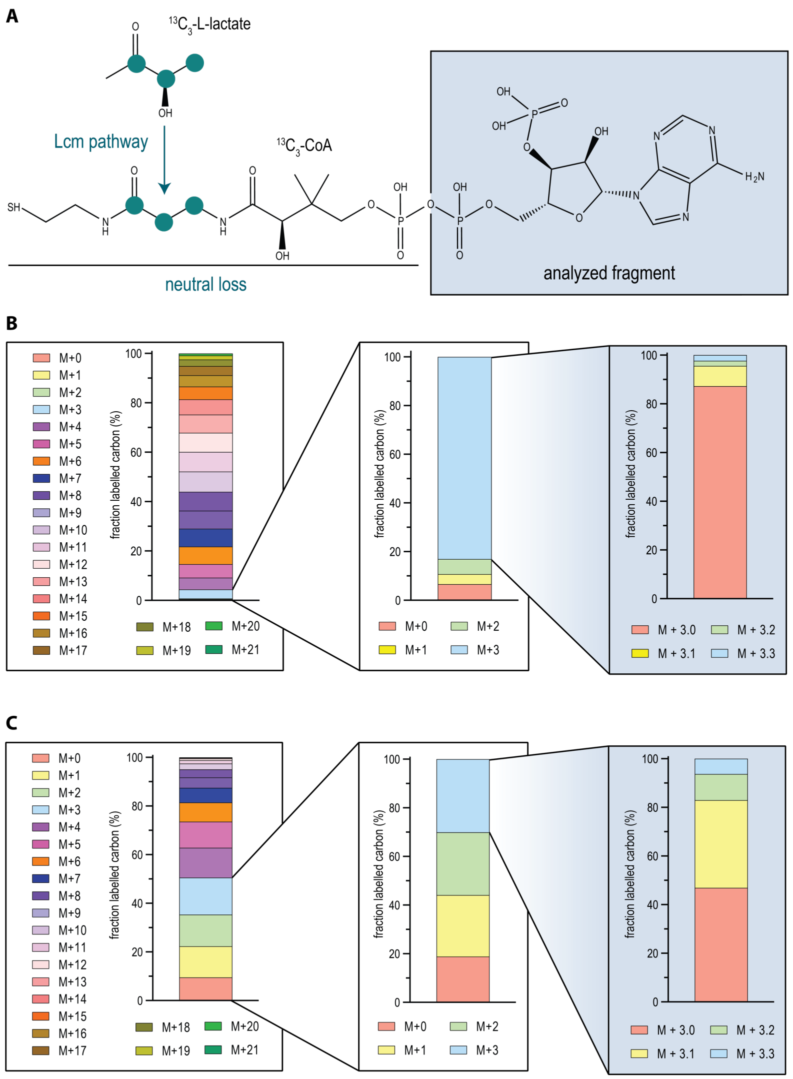

Supplementary Figure 8. Isotopic labelling patterns for the untargeted analyses of free CoA. A Δ*lldD* strain was used as control, and the β-alanine auxotroph with pBBR1-Lcm and chromosomal-rbsA-*abfT* expression was used. **A)** Expected isotopic labelling distribution in free CoA from β-alanine auxotroph growing via the Lcm pathway. ^{13}C atoms should only be present in the neutral loss moiety of CoA during targeted MS/MS analysis. **B)** Labelling patterns in free CoA from a β-alanine auxotrophic strain expressing the Lcm pathway that was grown in minimal medium with 20 mM glycerol + 1 mM IPTG + 1 µM B12 + 20 mM $^{13}C_3$-L-lactate. Left graph: presentation of all masses until M+21 (equivalent to the number of carbons in CoA) determined by untargeted mass analysis. Middle graph: presentation of the masses m+0 to m+3, determined by untargeted mass analysis. Right graph: Mass distribution in the analyzed fragment of three-fold labelled CoA, determined by fragmentation and targeted mass analysis. See methods section for parameters used for CoA fragmentation. The annotation for the right graph in panel C denotes three labelled carbons in the entire molecule (3.X) and the number of labelled carbons in the analyzed fragment (e.g., one labelled carbon in the analyzed fragment represented as 3.1). Mean values of technical triplicates were used to determine fractions. **C)** Labelling patterns in free CoA from the Δ*lldD* control strain grown with 20 mM glycerol + 1 mM IPTG + 1 µM B12 + 20 mM $^{13}C_3$-L-lactate. Graph distribution from left to right is coherent to (B). Mean values of technical triplicates were used to determine fractions. Source data are provided as Source Data file.

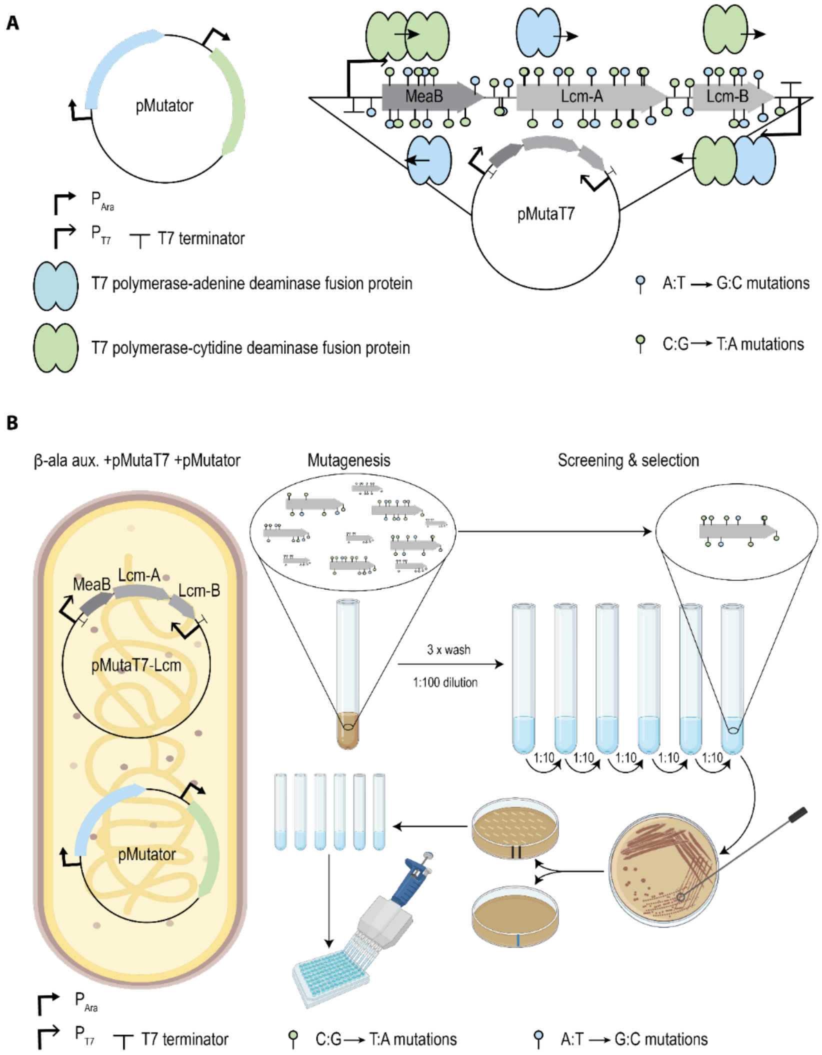

Supplementary Figure 9. Workflow for the generation of improved Lcm variants by mutagenesis employing the eMutaT7 system. A) Schematic representation of the MutaT7 system for hypermutagenesis of the operon containing the chaperone MeaB and both Lcm subunits. **B)** The genes encoding Lcm (light grey) and the chaperone MeaB (dark grey) are expressed from an IPTG-inducible target plasmid where they are flanked by T7 promoters (pMutaT7). From the mutator plasmid, two individual fusion proteins are expressed, consisting of T7 polymerase and either cytidine deaminase (facilitating C (→ U) → T exchanges) or adenine deaminase (facilitating A → G mutations), respectively. Induction of the deaminase fusions should result in an increased mutagenesis rate on the T7-promoter-flanked genes[8]. Both constructs were introduced to the β-alanine aux. strain (chromosomal rbsA-*abfT*). Since the strain was only able to grow in selective medium without the mutator, mutagenesis was performed in LB with ampicillin (for target plasmid maintenance) and chloramphenicol (for mutator maintenance) by mutator expression induction overnight (10 mM L-arabinose). The cells were then washed and propagated several times in selective medium, before isolating single colonies on LB with ampicillin. Single colonies were restreaked to check for mutator presence (none carried the mutator plasmid). After confirming their growth in selective medium in tubes, they were compared to the ancestral strains in a plate reader experiment. This figure was created using Biorender.com (Created in BioRender. Schulz-Mirbach, H. (2023) BioRender.com/i89k495).

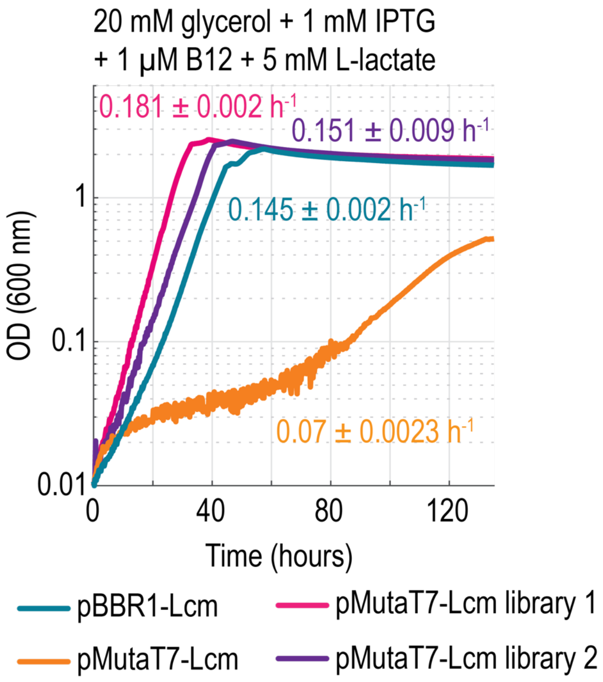

Supplementary Figure 10. The evolved pMutaT7-Lcm populations grow faster than previously characterized strains. Growth was tested in M9 medium with 20 mM glycerol + 1 mM IPTG + 1 µM B12 + 5 mM L-lactate. Tested strains are indicated by the legend. Source data are provided as Source Data file.

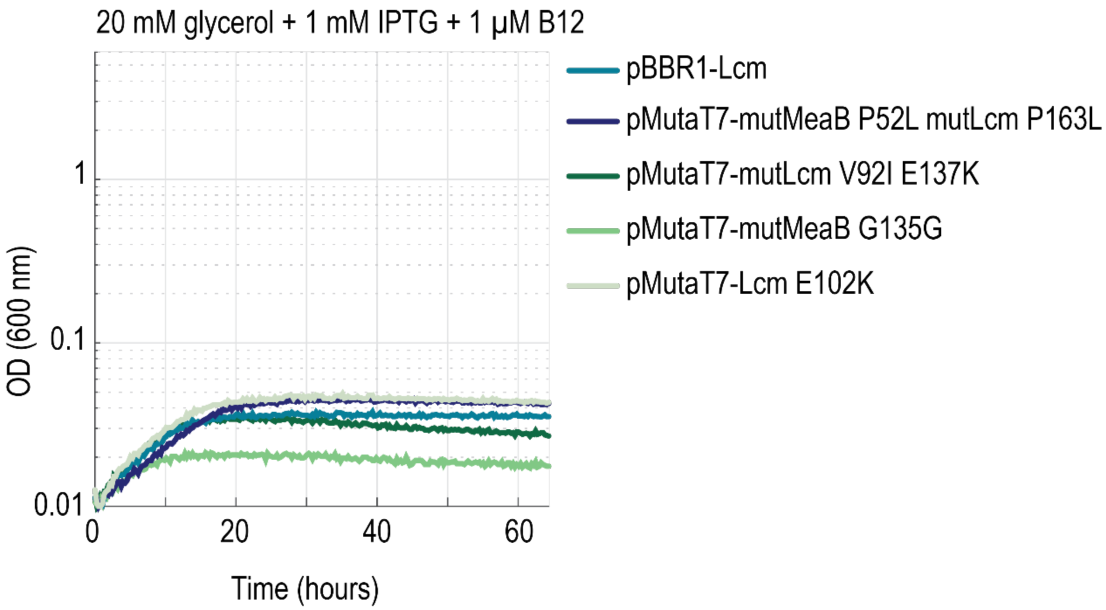

Supplementary Figure 11. Growth of all mutase dependent strains requires lactate. Shown are negative controls (no L-lactate) of the β-alanine auxotrophic strains with chromosomal-rbsA-abfT expressing the Lcm either from the original plasmid (pBBR1, teal curve), or mutated single copy MutaT7 target plasmids (dark blue and green lines) in Fig. 4C. Source data are provided as Source Data file.

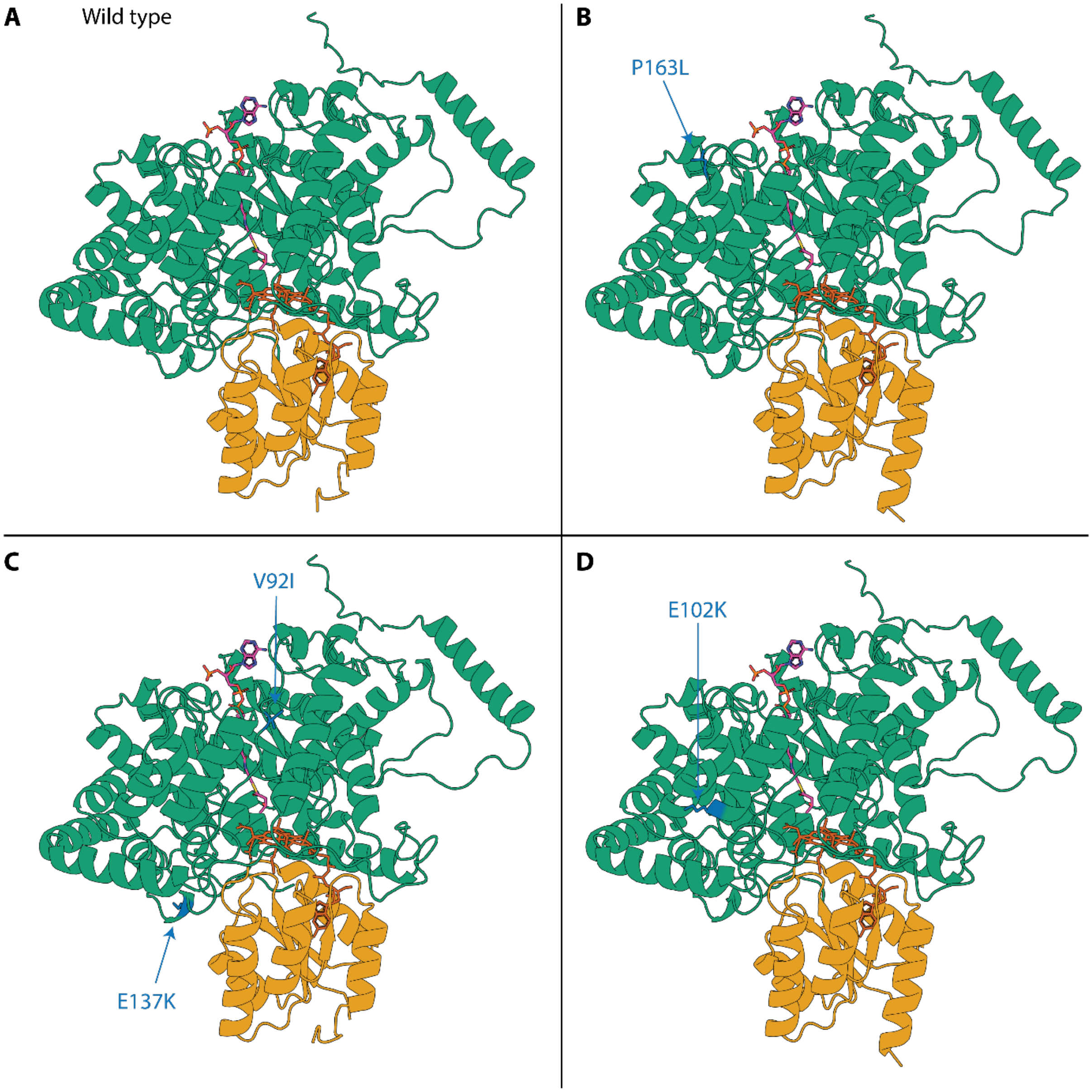

Supplementary Figure 12. AlphaFold2 predictions of all Lcm variants. None of the obtained mutations (highlighted blue, indicated by blue arrows) directly points towards the substrate or changes the predicted enzyme structure. The large subunit is shown in green, the small subunit in yellow. Mutations are highlighted in blue. Coenzyme B12 (dark orange) as well as 2-hydroxyisobutyryl-CoA (pink) were modeled into the structure based on the *A. tertiaricarbonis* Hcm structure (PDB: 4r3u). **A)** Predicted structure of the wild-type Lcm. **B)** Predicted structure of the Lcm variant P163L. **C)** Predicted structure of the Lcm variant V92I E137K. **D)** Predicted structure of the Lcm variant E102K.

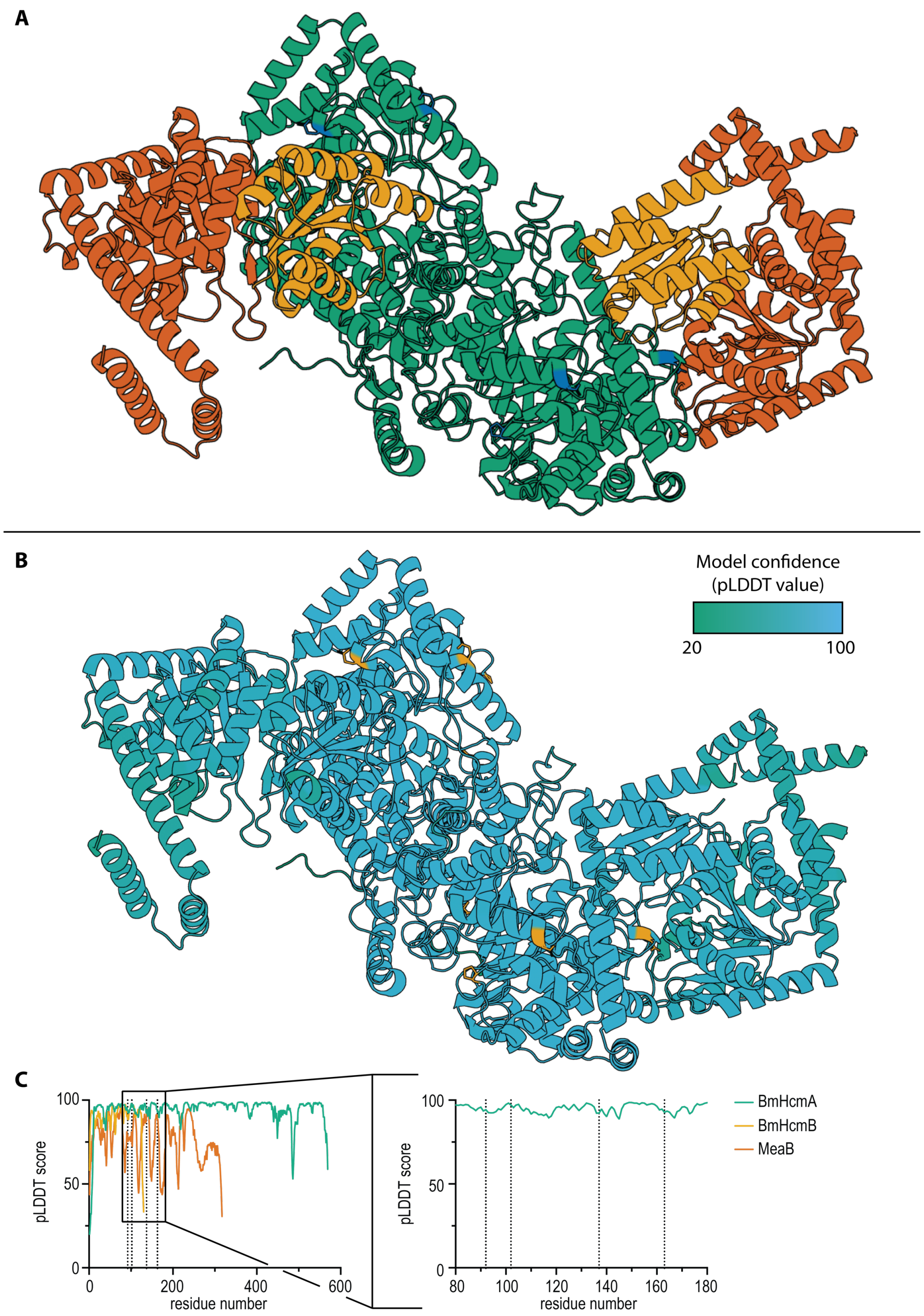

Supplementary Figure 13: AlphaFold Multimer predictions (based on AlphaFold2) of the Lcm tetramer and the chaperone MeaB based on the *A. tertiaricarbonis* Hcm structure (PDB: 4r3u). A) Predicted structure of the Lcm tetramer (two large subunits shown in green, with the four mutations obtained in this study highlighted in blue, and two small subunits in yellow) together with the chaperone MeaB (orange). None of the obtained mutations are located at interaction interfaces. **B)** The structure shown in panel (A) colored according to modelling confidence (pLDDT value), with obtained mutations displayed in yellow. **C)** Model confidence for the Lcm (large subunit in green, small subunit in yellow) and MeaB (orange). Vertical dotted lines indicate the positions of mutated/evolved residues identified in this study. None of the mutations are in low-confidence regions. Source data are provided as Source Data file.

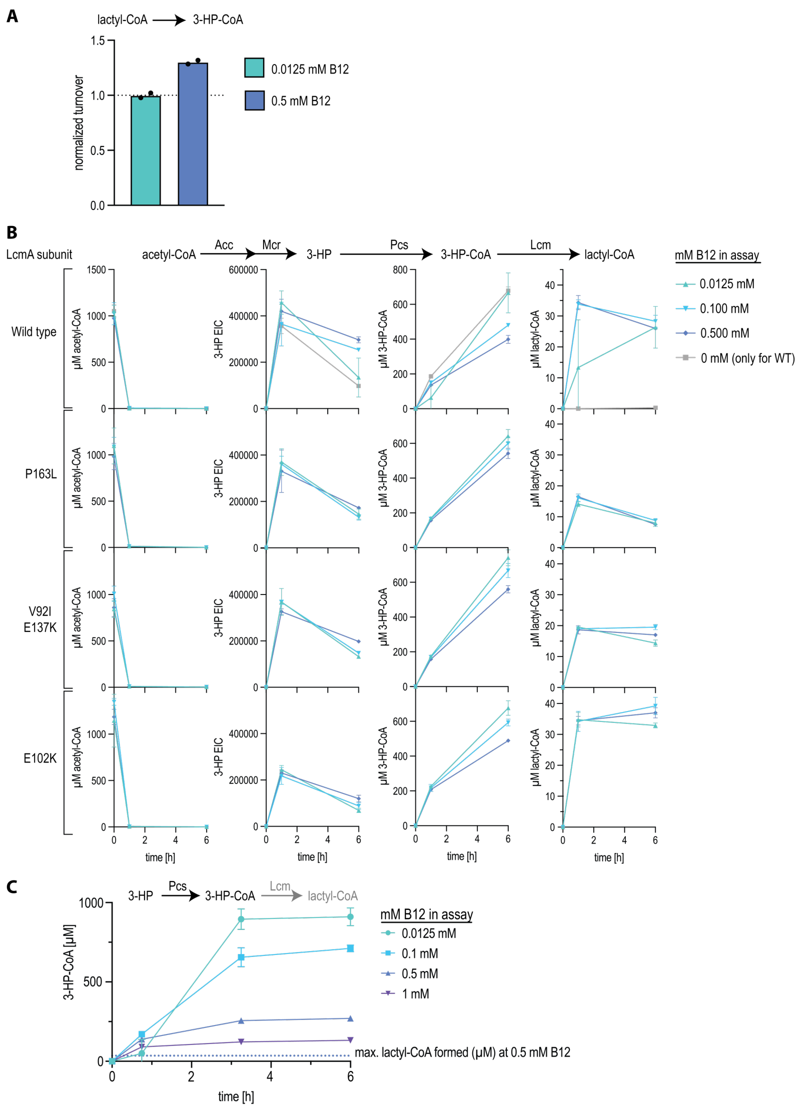

Supplementary Figure 14: B_{12}-dependence of Lcm and Pcs activity. A) Activity of the Lcm (wild-type variant) in presence of 12.5 or 500 µM B_{12}. 1 mM 3-HP-CoA was added to the reaction, the assay was performed as described in the main text (Methods), but with 100 mM HEPES (instead of sodium phosphate buffer). Due to interference of high B_{12} concentrations with the photometric assay readout, the 500 µM B_{12} assay was performed with 100 µM NADPH instead of 0.25 mM to decrease the overall absorbance. **B)** Lcm module intermediate formation from 1 mM acetyl-CoA in the Lcm module with Pcs over time with 12.5 µM B_{12} (turquoise), 100 µM B_{12} (light blue) or 500 µM B_{12} (dark blue). The Lcm variant used is indicated on the left of each panel. The metabolite is indicated above each column. Acetyl-CoA, 3-HP-CoA and lactyl-CoA absolute concentrations are shown, for 3-HP the extracted ion chromatogram is shown. Measurements were conducted in triplicates, the mean and standard deviation are shown. **C)** 3-HP-CoA formation from 1 mM 3-HP by the Pcs in presence of the wild-type mutase and different B_{12} concentrations (indicated by the legend to the right). A maximum of 34.8 µM lactyl-CoA formed by the wild-type mutase could be detected in the assay. Note that lactyl-CoA formation does not make up for the severe decrease in formed 3-HP-CoA, which indicates inhibition of the Pcs by high amounts of B_{12}. Mean values of n=3 are shown with standard deviation in all panels. Source data are provided as Source Data file.

Supplementary Table 1. Discovered natural carbon fixation pathways and their metabolic products

Pathway Name	Cyclic / Linear	O_2-tolerance	Carbon Input	Initial product	Initial *Central Metabolic* Product	Reference
reductive acetyl-CoA pathway (Wood-Ljungdahl Pathway)	Linear	O_2-sensitive	CO_2 (2x) / reduced C1	acetyl-CoA	**acetyl-CoA**	reviewed in [9]
reductive glycine pathway (glycine reductase variant)	Linear	O_2-sensitive	CO_2 (2x) / reduced C1	glycine	**acetyl-CoA**	[10]
reductive TCA Cycle	Cyclic	O_2-sensitive	CO_2 (2x)	acetyl-CoA	**acetyl-CoA**	reviewed in [9]; [11]
reverse oxidative TCA Cycle	Cyclic	O_2-tolerant	CO_2 (2x)	acetyl-CoA	**acetyl-CoA**	[12,13]
Dicarboxylate/ 4-hydroxybutyrate Cycle	Cyclic	O_2-sensitive	CO_2, HCO_3^-	acetyl-CoA	**acetyl-CoA**	reviewed in [9]
3-hydroxypropionate/ 4-hydroxybutyrate Cycle	Cyclic	O_2-tolerant	HCO_3^- (2x)	acetyl-CoA	**acetyl-CoA**	reviewed in [9]
reductive pentose phosphate pathway (Calvin-Benson-Bassham Cycle)	Cyclic	O_2 side-activity	CO_2 (3x)	3-phospho-glycerate	**autocatalytic (sugar phosphates)**	reviewed in [9]
3-hydroxypropionate Bicycle	Cyclic	O_2-tolerant	HCO_3^- (3x)	glyoxylate	**pyruvate**	reviewed in [9]

Supplementary Table 2. Parameter settings for the LC-MS based quantification of CoA esters.

Name	Precurser Ion	Product Ion	Collision energy [V]	Fragmentor Voltage [V]	Cell Accelorator Voltage [V]	Dwell time [msec]	Polarity
Malonyl-CoA	854.1	428.1	30	380	5	160	positive
Malonyl-CoA	854.1	347.1	37	380	5	160	Positive
Lactyl-CoA	840.3	428.1	30	380	5	160	Positive
Lactyl-CoA	840.3	333.4	33	380	5	160	Positive
3-Hydroxypropionyl-CoA	840.3	428.1	30	380	5	160	Positive
3-Hydroxypropionyl-CoA	840.3	333.4	33	380	5	160	Positive
Acetyl-CoA	810.1	428.1	35	380	5	160	Positive
Acetyl-CoA	810.1	302.2	35	380	5	160	positive

Supplementary Table 3. Parameter settings for the LC-MS based quantification of lactate and 3-HP

Name	Precurser Ion	Product Ion	Collision energy [V]	Fragmentor Voltage [V]	Cell Accelorator Voltage [V]	Dwell time [msec]	Polarity
Lactate	89.2	89.2	0	380	5	180	Negative
Lactate	89.2	71.3	10	380	5	180	Negative
3-Hydroxypropionate	89.2	89.2	0	380	5	180	Negative
3-Hydroxypropionate	89.2	59	7	380	5	180	Negative

Supplementary Table 4. m/z values used for the untargeted ^{13}C isotopic labelling analysis of CoA by liquid chromatography mass spectrometry (LC-MS)**.**

Name	Formula	Formula [M+H]+	m/z [M+H]+
CoA [M+00]	[13C]0C21H36N7O16P3S	[13C]0C21H37N7O16P3S+	768.1225
CoA [M+01]	[13C]1C20H36N7O16P3S	[13C]1C20H37N7O16P3S+	769.1258
CoA [M+02]	[13C]2C19H36N7O16P3S	[13C]2C19H37N7O16P3S+	770.1292
CoA [M+03]	[13C]3C18H36N7O16P3S	[13C]3C18H37N7O16P3S+	771.1326
CoA [M+04]	[13C]4C17H36N7O16P3S	[13C]4C17H37N7O16P3S+	772.1359
CoA [M+05]	[13C]5C16H36N7O16P3S	[13C]5C16H37N7O16P3S+	773.1393
CoA [M+06]	[13C]6C15H36N7O16P3S	[13C]6C15H37N7O16P3S+	774.1426
CoA [M+07]	[13C]7C14H36N7O16P3S	[13C]7C14H37N7O16P3S+	775.146
CoA [M+08]	[13C]8C13H36N7O16P3S	[13C]8C13H37N7O16P3S+	776.1493
CoA [M+09]	[13C]9C12H36N7O16P3S	[13C]9C12H37N7O16P3S+	777.1527
CoA [M+10]	[13C]10C11H36N7O16P3S	[13C]10C11H37N7O16P3S+	778.156
CoA [M+11]	[13C]11C10H36N7O16P3S	[13C]11C10H37N7O16P3S+	779.1594
CoA [M+12]	[13C]12C9H36N7O16P3S	[13C]12C9H37N7O16P3S+	780.1627
CoA [M+13]	[13C]13C8H36N7O16P3S	[13C]13C8H37N7O16P3S+	781.1661
CoA [M+14]	[13C]14C7H36N7O16P3S	[13C]14C7H37N7O16P3S+	782.1695
CoA [M+15]	[13C]15C6H36N7O16P3S	[13C]15C6H37N7O16P3S+	783.1728
CoA [M+16]	[13C]16C5H36N7O16P3S	[13C]16C5H37N7O16P3S+	784.1762
CoA [M+17]	[13C]17C4H36N7O16P3S	[13C]17C4H37N7O16P3S+	785.1795
CoA [M+18]	[13C]18C3H36N7O16P3S	[13C]18C3H37N7O16P3S+	786.1829
CoA [M+19]	[13C]19C2H36N7O16P3S	[13C]19C2H37N7O16P3S+	787.1862
CoA [M+20]	[13C]20C1H36N7O16P3S	[13C]20C1H37N7O16P3S+	788.1896
CoA [M+21]	[13C]21C0H36N7O16P3S	[13C]21C0H37N7O16P3S+	789.1929

Supplementary Table 5. m/z values used for the targeted ^{13}C isotopic labelling analysis of CoA by liquid chromatography mass spectrometry (LC-MS)**.**

Name	Precurser Ion	Product Ion	Collision energy [V]	Fragmentor Voltage [V]	Cell Accelorator Voltage [V]	Dwell time [msec]	Polarity
CoA [M+0.0]	768.12	428	31	380	5	100	Positive
CoA [M+1.0]	769.12	428	31	380	5	100	Positive
CoA [M+1.1}	769.12	429	31	380	5	100	Positive
CoA [M+2.0]	770.12	428	31	380	5	100	Positive
CoA [M+2.1]	770.12	429	31	380	5	100	Positive
CoA [M+2.2]	770.12	430	31	380	5	100	Positive
CoA [M+3.0]	771.12	428	31	380	5	100	Positive
CoA [M+3.1]	771.12	429	31	380	5	100	Positive
CoA [M+3.2]	771.12	430	31	380	5	100	Positive
CoA [M+3.3]	771.12	431	31	380	5	100	Positive

Supplementary references

1. Beber, M. E. *et al.* eQuilibrator 3.0: a database solution for thermodynamic constant estimation. *Nucleic Acids Res.* **50**, D603–D609 (2022).
2. Löwe, H. & Kremling, A. In-Depth Computational Analysis of Natural and Artificial Carbon Fixation Pathways. *BioDesign Res.* **2021**, 1–23 (2021).
3. Luo, S. *et al.* A cell-free self-replenishing CO_2-fixing system. *Nat. Catal.* **5**, 154–162 (2022).
4. Bar-Even, A., Noor, E., Lewis, N. E. & Milo, R. Design and analysis of synthetic carbon fixation pathways. *Proc. Natl. Acad. Sci.* **107**, 8889–8894 (2010).
5. Zarzycki, J. & Fuchs, G. Coassimilation of organic substrates via the autotrophic 3-hydroxypropionate bi-cycle in *Chloroflexus aurantiacus*. *Appl. Environ. Microbiol.* **77**, 6181–6188 (2011).
6. Edgar, R. C. MUSCLE: multiple sequence alignment with high accuracy and high throughput. *Nucleic Acids Res.* **32**, 1792–1797 (2004).
7. Price, M. N., Dehal, P. S. & Arkin, A. P. FastTree 2 – Approximately Maximum-Likelihood Trees for Large Alignments. *PLoS ONE* **5**, e9490 (2010).
8. Seo, D., Koh, B., Eom, G.-E., Kim, H. W. & Kim, S. A dual gene-specific mutator system installs all transition mutations at similar frequencies *in vivo*. *Nucleic Acids Res.* **51**, e59 (2023).
9. Bar-Even, A., Noor, E. & Milo, R. A survey of carbon fixation pathways through a quantitative lens. *J. Exp. Bot.* **63**, 2325–2342 (2012).
10. Sánchez-Andrea, I. *et al.* The reductive glycine pathway allows autotrophic growth of *Desulfovibrio desulfuricans*. *Nat. Commun.* **11**, 5090 (2020).
11. Evans, M. C., Buchanan, B. B. & Arnon, D. I. A new ferredoxin-dependent carbon reduction cycle in a photosynthetic bacterium. *Proc. Natl. Acad. Sci. U. S. A.* **55**, 928–934 (1966).
12. Mall, A. *et al.* Reversibility of citrate synthase allows autotrophic growth of a thermophilic bacterium. *Science* **359**, 563–567 (2018).
13. Nunoura, T. *et al.* A primordial and reversible TCA cycle in a facultatively chemolithoautotrophic thermophile. *Science* **359**, 559–563 (2018).
14. Jensen, S. I., Lennen, R. M., Herrgård, M. J. & Nielsen, A. T. Seven gene deletions in seven days: Fast generation of *Escherichia coli* strains tolerant to acetate and osmotic stress. *Sci. Rep.* **5**, 17874 (2016).
15. Braatsch, S., Helmark, S., Kranz, H., Koebmann, B. & Jensen, P. R. *Escherichia coli* strains with promoter libraries constructed by Red/ET recombination pave the way for transcriptional fine-tuning. *BioTechniques* **45**, 335–337 (2008).
16. Schulz-Mirbach, H. *et al.* On the flexibility of the cellular amination network in *E. coli*. *eLife* **11**, e77492 (2022).
17. Datsenko, K. A. & Wanner, B. L. One-step inactivation of chromosomal genes in *Escherichia coli* K-12 using PCR products. *Proc. Natl. Acad. Sci.* **97**, 6640–6645 (2000).

18. Wenk, S., Yishai, O., Lindner, S. N. & Bar-Even, A. An Engineering Approach for Rewiring Microbial Metabolism. in *Methods in Enzymology* vol. 608 329–367 (Elsevier, 2018).

19. Zelcbuch, L. *et al.* Spanning high-dimensional expression space using ribosome-binding site combinatorics. *Nucleic Acids Res.* **41**, e98 (2013).

20. Rohde, M.-T., Tischer, S., Harms, H. & Rohwerder, T. Production of 2-Hydroxyisobutyric Acid from Methanol by *Methylobacterium extorquens* AM1 Expressing (*R*)-3-Hydroxybutyryl Coenzyme A-Isomerizing Enzymes. *Appl. Environ. Microbiol.* **83**, e02622-16 (2017).

21. Park, H. & Kim, S. Gene-specific mutagenesis enables rapid continuous evolution of enzymes in vivo. *Nucleic Acids Res.* **49**, e32 (2021).

22. Scheffen, M. *et al.* A new-to-nature carboxylation module to improve natural and synthetic CO_2 fixation. *Nat. Catal.* **4**, 105–115 (2021).

23. Weichler, M.-T. *et al.* Thermophilic Coenzyme B12-Dependent Acyl Coenzyme A (CoA) Mutase from *Kyrpidia tusciae* DSM 2912 Preferentially Catalyzes Isomerization of (R)-3-Hydroxybutyryl-CoA and 2-Hydroxyisobutyryl-CoA. *Appl. Environ. Microbiol.* **81**, 4564–4572 (2015).

24. McLean, R. *et al.* Exploring alternative pathways for the in vitro establishment of the HOPAC cycle for synthetic CO_2 fixation. *Sci. Adv.* **9**, eadh4299 (2023).

25. Bernhardsgrütter, I. *et al.* The multicatalytic compartment of propionyl-CoA synthase sequesters a toxic metabolite. *Nat. Chem. Biol.* **14**, 1127–1132 (2018).
