# Supplementary material for: New-to-nature CO2-dependent acetyl-CoA assimilation enabled by an engineered B12-dependent acyl-CoA mutase
Source: Nat Commun. 2024 Nov 26;15:10235. doi: 10.1038/s41467-024-53762-9 (PMC11599936; doi:10.1038/s41467-024-53762-9)
Supplement: Supplementary file 3 — Description of Additional Supplementary Files [file 41467_2024_53762_MOESM3_ESM.pdf]

**Description of Additional Supplementary Files**

---

**File Name:** Supplementary Data 1

**Description:** Comparison of the energetic costs and Max-Min-driving force (MDF) of the presented acetyl-CoA assimilation routes.

---

**File Name:** Supplementary Data 2

**Description:** Strains and plasmids used in this study.

---

**File Name:** Supplementary Data 3

**Description:** Oligonucleotide primers used. ‘KO’ primers were used for amplification of the CapR cassette from pKD3 (overlap with CapR sequence marked in bold) with 50 bp overhangs homologous to regions upstream and downstream of the target locus (Purpose 1). ‘KO_Ver’-primers were used to verify successful by CapR resistance cassette insertion as well as flippase mediated cassette removal (Purpose 2).

---

**File Name:** Supplementary Data 4

**Description:** Sequences of synthesized genes used in this study with NsiI and XbaI restriction sites, His-tag and linker.
